# A targeted e-learning approach for keeping universities open during the COVID-19 pandemic while reducing student physical interactions

**DOI:** 10.1371/journal.pone.0249839

**Published:** 2021-04-08

**Authors:** Sing Chen Yeo, Clin K. Y. Lai, Jacinda Tan, Joshua J. Gooley

**Affiliations:** 1 Neuroscience and Behavioural Disorders Programme, Duke-NUS Medical School, Singapore, Singapore; 2 Institute for Applied Learning Sciences and Educational Technology, National University of Singapore, Singapore, Singapore; Drexel University School of Public Health, UNITED STATES

## Abstract

The COVID-19 pandemic led to widespread closure of universities. Many universities turned to e-learning to provide educational continuity, but they now face the challenge of how to reopen safely and resume in-class learning. This is difficult to achieve without methods for measuring the impact of school policies on student physical interactions. Here, we show that selectively deploying e-learning for larger classes is highly effective at decreasing campus-wide opportunities for student-to-student contact, while allowing most in-class learning to continue uninterrupted. We conducted a natural experiment at a large university that implemented a series of e-learning interventions during the COVID-19 outbreak. The numbers and locations of 24,000 students on campus were measured over a 17-week period by analysing >24 million student connections to the university Wi-Fi network. We show that daily population size can be manipulated by e-learning in a targeted manner according to class size characteristics. Student mixing showed accelerated growth with population size according to a power law distribution. Therefore, a small e-learning dependent decrease in population size resulted in a large reduction in student clustering behaviour. Our results suggest that converting a small number of classes to e-learning can decrease potential for disease transmission while minimising disruption to university operations. Universities should consider targeted e-learning a viable strategy for providing educational continuity during periods of low community disease transmission.

## Introduction

The coronavirus disease 2019 (COVID-19) has had enormous socioeconomic impact [1]. Over the course of 14 months (as of March 17, 2021), more than 120 million COVID-19 cases were confirmed, resulting in 2.67 million deaths across 192 countries/regions [2]. The severe acute respiratory syndrome coronavirus 2 (SARS-CoV-2) that causes COVID-19 is spread by virus-containing droplets released when an infected person speaks, sneezes, or coughs [3], or by aerosol particles produced by breathing or talking [4]. The spread of infection can be slowed by public health measures that reduce person-to-person contact. Nonpharmaceutical interventions that include restricted travel, staying at home, and physical distancing can delay and flatten the peak of COVID-19 cases to avoid the overwhelming of medical services [5–7]. Nonpharmaceutical interventions therefore play a critical role in controlling the spread of disease until effective vaccines or drugs are widely available [8].

School closure is a key strategy for controlling the spread of infectious diseases [8–10]. Many epidemiological studies have shown that school closure can reduce the transmission of seasonal and pandemic influenza among school-aged children [11]. This effect is sometimes reversed when schools re-open, suggesting a causal role of school closure in reducing the incidence of influenza. The extent to which school closure can mitigate the spread of COVID-19 and other coronaviruses is unclear [12]. Empirical and modelling studies show that closing schools and universities can suppress COVID-19 transmission when combined with other nonpharmaceutical interventions [6, 7, 13]. In the spring of 2020, closure of schools in the United States was associated with a decline in the incidence of COVID-19 and mortality [14]. Conversely, reopening of colleges and universities in the fall semester was associated with a surge in confirmed SARS-CoV-2 infections [15, 16]. These studies suggest that closing schools can potentially reduce COVID-19 transmission but the effects are difficult to isolate because other nonpharmaceutical interventions were enacted concurrently. Protracted closure of schools and universities is a controversial policy because of high social and economic costs.

The COVID-19 pandemic resulted in an unprecedented number of school and university closures, affecting over 1.2 billion learners worldwide [17]. Consequently, a massive shift from classroom learning to distance learning occurred [18]. This created great strain on educational institutions which function not only as places of learning, but also as major employers and drivers of local economies. It is therefore important to consider less disruptive interventions to ensure educational continuity [19]. Many higher education providers transitioned to a hybrid learning approach comprising a combination of face-to-face and online learning methods [15, 20]. However, there is a major knowledge gap in how student mixing patterns on campus are affected by school policies enacted during the disease outbreak.

**Fig 1:**
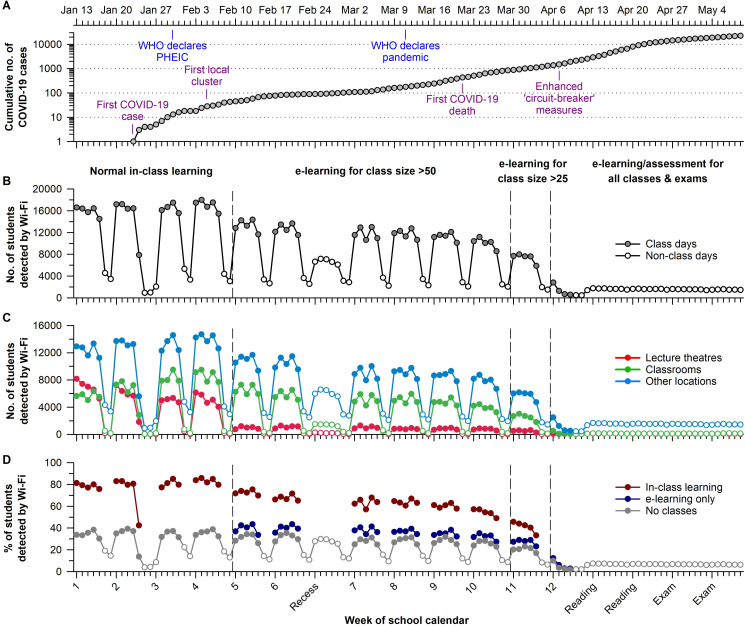
E-learning interventions decreased the number of students detected on campus during the COVID-19 outbreak. (**A**) The timeline of COVID-19 cases and events in Singapore is shown for the second semester of the 2019/20 school year at the National University of Singapore (NUS). Each e-learning intervention was associated with a decrease in the daily number of students who connected to the NUS Wi-Fi network, assessed (**B**) campus-wide and (**C**) for different types of locations on campus. (**D**) The daily percentage of students detected by Wi-Fi was about two-fold greater in students with at least one class conducted by in-class learning, as compared with students with e-learning only or no scheduled class. In panels **C** and **D**, open circles indicate non-class days. COVID-19, Coronavirus Disease 2019; WHO, World Health Organisation; PHEIC, Public Health Emergency of International Concern.

Here, we conducted a natural experiment to test the impact of implementing e-learning measures on student population dynamics during the COVID-19 outbreak. The research was conducted at the National University of Singapore (NUS), which is the largest university by student enrolment in Singapore. The short-term goal of our research was to help NUS leaders make evidence-based decisions on how to resume in-class learning during the pandemic. The broader scientific goal was to determine the underlying mathematical relationships between students’ daily population size and mixing patterns on campus, which has implications for disease transmission risk. In line with the national public health response during the COVID-19 outbreak, NUS adopted nonpharmaceutical interventions that aimed to reduce risk of SARS-CoV-2 transmission (**S1 Table**). Normal in-class learning took place during the first 4 weeks of the semester, coinciding with the first imported case of COVID-19 (**Fig 1A**). Shortly afterward the first local transmission of COVID-19 was identified. This escalated the nationwide pandemic response and prompted NUS to implement e-learning over the next several weeks for all classes with >50 students. As the number of COVID-19 cases continued to climb in Singapore and globally as a pandemic, NUS implemented e-learning for all classes with >25 students. One week later, nationwide ‘enhanced circuit-breaker’ measures were announced, which led to the suspension of all in-class learning.

The multi-phased transition to e-learning during the COVID-19 outbreak provided a unique opportunity to investigate student mixing patterns on campus. Previous studies have shown that social interactions and their products show super-linear growth with population size [21–25]. Hence, interventions that reduce the number of students by a small amount might decrease substantially the potential for person-to-person contact and disease transmission. Here, we tested the hypothesis that student population size on campus can be manipulated by e-learning in a targeted manner to produce a large, non-linear drop in opportunities for student mixing. This hypothesis was tested by analysing >24 million student connections to the NUS Wi-Fi network, comprising several thousand Wi-Fi access points across campus. These Wi-Fi connection data were used to investigate students’ spatiotemporal mixing patterns (i.e., connected to the same Wi-Fi access point at the same time) before and during the disease outbreak.

## Materials and methods

### Student data and ethics statement

Our study was performed using university archived data managed by the NUS Institute for Applied Learning Sciences and Educational Technology (ALSET). The ALSET Data Lake stores and links deidentified student data across different university units for the purpose of conducting educational analytics research [26]. Data tables in the ALSET Data Lake are anonymised by student tokens which map identifiable data to a hash string using a one-way function that does not allow recovery of the original data. The same student-specific tokens are represented across tables, allowing different types of data to be combined without knowing students’ identities. The data types used in our study included basic demographic information (age, sex, ethnicity, citizenship, year of matriculation), class enrolment information, and Wi-Fi connection metadata. Upon enrolling in the university, students provided written informed consent to the NUS Student Data Protection Policy, which explains that their personal data may be used for research including evaluating university policies. In accordance with NUS guidelines for educational research, students were not required to consent to the specific set of analyses in our study because the research involved retrospective analyses of de-identified data. The research was approved by the NUS Learning Analytics Committee on Ethics (LACE), which is a Departmental Ethics Review Committee (DERC) that oversees educational research that qualifies for exemption from review by the NUS Institutional Review Board (IRB). Analyses were performed and stored on the ALSET Data Lake data server in accordance with NUS data management policies for students’ personal data.

### Timeline of COVID-19 cases and university policies

The timeline of COVID-19 cases in Singapore was determined using daily situation reports published online by the Ministry of Health (MOH) [27, 28]. Nationwide alerts and policies regarding the public health response were taken from press releases available on the MOH website [29]. University policies enacted during the COVID-19 outbreak were compiled from circulars distributed to staff and students, and they are archived by the NUS Office of Safety, Health, and Environment [30].

### Student timetables and class size characteristics

Student data were analysed in the second semester of the 2018/19 and 2019/20 school years. This allowed us to compare student behaviour before and during the COVID-19 outbreak over an equivalent period (from January to May). Students’ class schedules and class sizes were derived from student enrolment data provided by the NUS Registrar’s Office. At NUS, students enrol in course modules, many of which are further divided into different lectures, class groups, tutorials, or laboratory sessions. We analysed data in students taking at least one module that required in-class learning (23,668 and 23,993 students in 2018/19 and 2019/20 school years). Data were excluded from students taking only fieldwork or project-based modules with no in-class component (2,722 and 3,240 students). Class size was defined as the number of students who were scheduled to meet in the same place for a given course module. The timing and location of classes were retrieved using the NUSMods application programming interface (https://api.nusmods.com/v2/). Timetable data were sorted for each school day of the semester to identify students with scheduled in-class learning. These data were also used to determine which classes were converted to e-learning based on class size. This allowed us to calculate the daily number of students with in-class learning, e-learning only, or no class.

### Wi-Fi connection data

Connections to the NUS Wi-Fi network are continually monitored by NUS Information Technology to evaluate and improve services provided to the university. The campus-wide wireless network comprises several thousand Wi-Fi access points and deploys different types of routers (*Cisco* Aironet 1142, 2702I and 2802I) and wireless protocols (802.11n 2.4 GHz, 802.11n 5 GHz, and 802.11ac 5 GHz). Each time that a person’s Wi-Fi enabled device associates with the NUS wireless network the transmission data are logged. Students’ Wi-Fi connection metadata were added daily to the ALSET Data Lake by a data pipeline managed by NUS Information Technology. Each data point included the tokenised student identity, the anonymised media access control (MAC) address used to identify the Wi-Fi enabled device of the student (e.g., smartphone, tablet, or laptop), the name and location descriptor of the Wi-Fi access point, and the start and end time of each Wi-Fi connection. The name and location descriptor usually carried information about the room or building in which the Wi-Fi access point was located. By cross-referencing these data with the known timing and location of classes, we categorised Wi-Fi access points into teaching facilities (lecture theatres or classrooms) and non-teaching facilities.

### Analyses of student mixing patterns

The Wi-Fi dataset comprised more than 24 million student connections to the wireless network over 2 semesters. Students’ Wi-Fi connection data were binned in 15-min intervals to reduce the size of the data, resulting in 11,328 epochs that spanned 118 days in each semester. In instances where students were connected to more than one Wi-Fi access point in the same epoch, they were assigned to the access point in which their Wi-Fi enabled device received the greatest volume of data (i.e., based on megabytes of data received). The resulting table of Wi-Fi connections and access points was used to derive time and location information for each student over the semester. This enabled us to count the daily number of students who connected to the Wi-Fi network, and the number of students who were connected to the same Wi-Fi access point within a 15-min epoch. The latter was used to examine student clustering behaviour. We defined a cluster as >25 students connected to the same Wi-Fi access point because of the high potential for spatiotemporal proximity and student interactions, and it aligned with the university’s e-learning policy prior to suspension of in-class learning (i.e., e-learning for class size >25). The duration of student clustering at each Wi-Fi access point was calculated as the sum of 15-min epochs with >25 students. Data were analysed using R statistical software (version 3.6.3) [31].

Geospatial clustering was visualised by plotting students’ data on a map of the NUS campus. The researchers did not have access to the geospatial coordinates for Wi-Fi access points. Therefore, general location information provided in the Wi-Fi metadata (e.g., name of the building or room) was used to determine manually the building locations. Using sources that included the official NUS campus map and venues listed on class timetables, we confirmed the geospatial coordinates for 80% of Wi-Fi access points. Georeferencing was performed by mapping Wi-Fi access points to vector point shapefiles representing individual buildings. The ESRI shapefiles required for mapping were obtained from the OpenStreetMap geodatabase for the region of Malaysia, Singapore, and Brunei (map tiles in the OpenStreetMap are licensed under CC BY-SA www.openstreetmap.org/copyright, © OpenStreetMap contributor). We used QGIS software (version 3.12.1) to edit the vector points and to insert names of Wi-Fi access points to the attribute table. Student clustering within each building was determined by pooling the duration of clustering across all Wi-Fi access points within the building. Subsequently, we merged the clustering duration data with the ESRI shapefiles using the “sf” package (version 0.9–0) [32] in the R software environment. The QGIS platform was then used to visualise student clustering for 124 buildings across the NUS campus. Buildings with incomplete Wi-Fi data and student hostels were excluded from the analysis.

The degree of Wi-Fi connection overlap for each student was determined by counting the number of unique students with whom he/she shared a Wi-Fi connection. Spatiotemporal overlap was determined for 4 representative weeks of the semester (weeks 4, 5, 11, 12). These time intervals captured the transition from normal in-class learning to e-learning for classes with >50 students (week 4 to 5), and the transition from e-learning for classes with >25 students to e-learning for all classes (week 11 to 12). The decision to focus on these temporal windows was driven by practical reasons related to computing resources required to analyse the data. Effects of e-learning on student network structure were visualised using the “igraph” package [33] (version 1.2.5) with the force-directed layout algorithm (layout_with_fr) in the R software environment. The degree of Wi-Fi connection overlap was plotted for 100 randomly selected participants to help illustrate changes in student network structure associated with each e-learning transition. We performed 20 iterations to confirm that results for the first group of 100 randomly selected participants were representative.

Student clustering behaviour on school days was modelled as a function of daily student population size using a power law scaling equation: *y* = *aN^β^*. In this equation, *y* is the measure of student mixing (e.g., number of Wi-Fi access points with a student cluster, duration of student clustering, or students’ degree of Wi-Fi connection overlap with other students); *a* is a constant; *N* is the daily population size estimated by the number of students who connected to the NUS Wi-Fi network; and the exponent *β* reflects the underlying dynamics (e.g., hierarchical structure, social networks, and infrastructure) of the university ecosystem. We considered other mathematical functions, including exponential and hyperbolic equations, but they did not fit as well to the data. Variables that show power law scaling are linearly related when each variable is logarithmically transformed. We therefore took the natural logarithm of each pair of variables (i.e., the student mixing variable and daily population size) and performed linear regression to confirm the expected linear relationship. The coefficient of determination (R^2^ value) was used to evaluate goodness-of-fit for the regression model. Modelling and regression analyses were performed using Sigmaplot software (Version 14; Systat Software, Inc) and R statistical software.

## Results

During the school semester in which the COVID-19 pandemic occurred, there were about 24,000 undergraduate students who were enrolled in course modules with in-class learning (**S2 Table**). In the early part of the semester when normal in-class learning took place, there were about 16,500 students per school day who connected to the NUS Wi-Fi network (**Fig 1B**). The only notable exception was the eve of the Chinese New Year holiday, in which the number of students detected by Wi-Fi dropped by about half. After the transition to e-learning for classes with >50 students, there was a 30% decrease in the daily number of students detected on campus. Wi-Fi connections decreased sharply in lecture theatres and moderately in classrooms and non-teaching facilities (**Fig 1C**). The number of students detected by Wi-Fi dropped by an additional 25% after e-learning was implemented for classes with >25 students. Once e-learning was implemented for all classes and exams, the daily population size fell below 1,800 students until the end of the semester. These findings contrast with results from the previous academic year in which the daily number of students detected by Wi-Fi on school days was stable across the semester (**S1 Fig**).

Effects of e-learning on the number of students on campus were determined by students’ class sizes and schedules. The transition to e-learning for classes with >50 or >25 students impacted a small proportion of total classes (9% and 19%, respectively) but these classes had high student enrolment (**S2 Fig**). Therefore, most students had at least one class that was converted to e-learning. On a typical school day, nearly 18,000 students had a scheduled class compared with 6,000 students with no class (**S2 Fig**). Due to heterogeneity in students’ timetables, the transition to e-learning resulted in a subset of students each day with classes delivered only by e-learning. This subset corresponded to about 5,000 and 9,000 students per day during periods when e-learning was implemented for classes with >50 students and >25 students, respectively. Students with e-learning only were detected on campus at about the same rate as students who had no class (about 35–40%) (**Fig 1D**). In contrast, students with in-class learning were detected at nearly twice the rate (about 60–80%) as students with e-learning only or no class. Linear regression analysis showed that 91% of the variance in the daily number of students detected on campus was explained by the number of students with in-class learning (**S3 Fig**).

**Fig 2:**
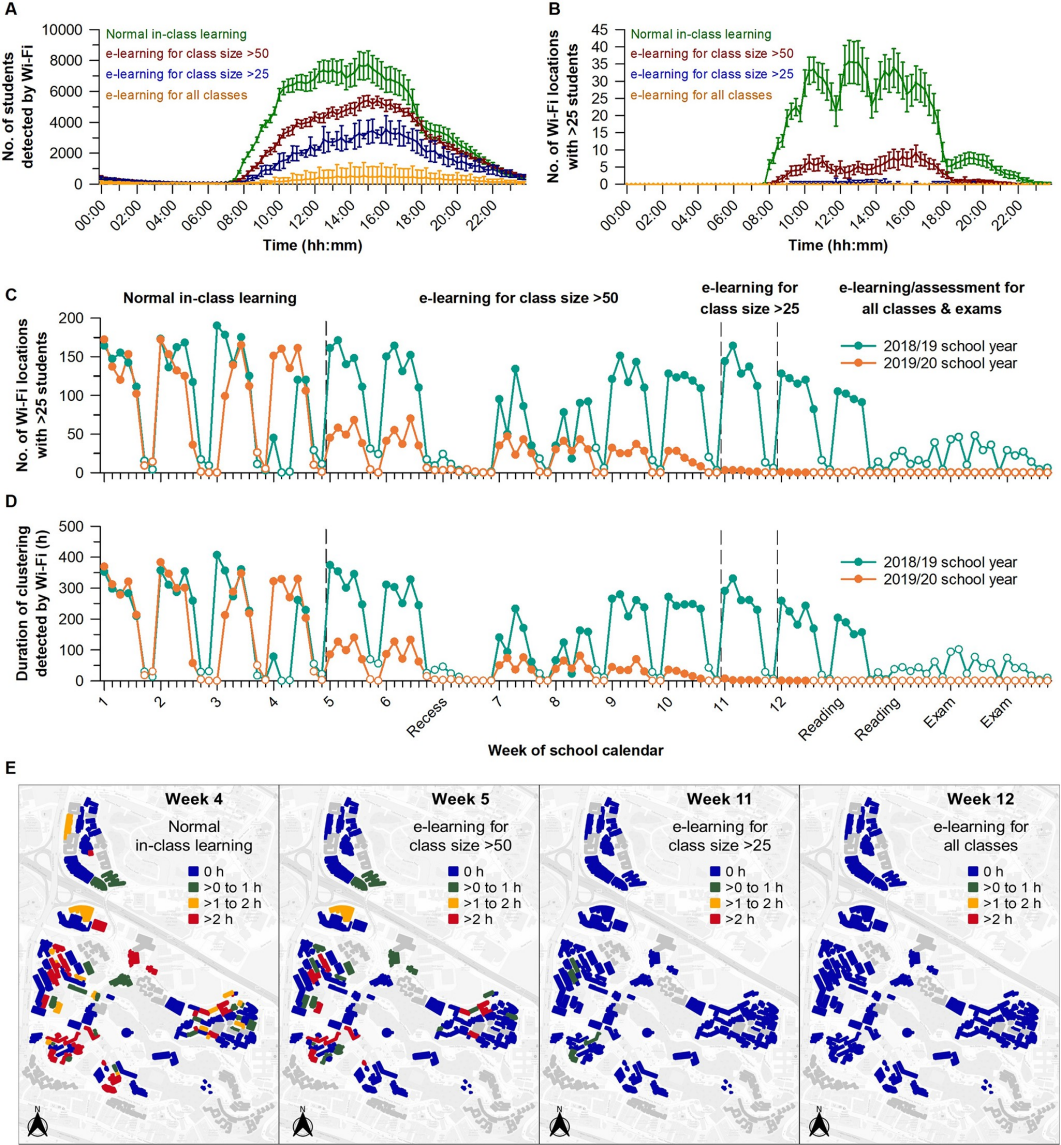
E-learning interventions reduced student clustering during the COVID-19 outbreak. Each e-learning intervention was associated with (**A**) a decrease in the daily rhythm in students detected on campus, and (**B**) a flattening in the daily time course of locations with >25 students connected to the same Wi-Fi access point. E-learning measures during the disease outbreak (2019/20 school year) were effective at decreasing (**C**) the daily number of Wi-Fi locations with a student cluster, and (**D**) the duration of clustering at these sites, as compared with the prior academic year with normal in-class learning (2018/19 school year). (**E**) The daily duration of student clustering in campus buildings decreased as more stringent e-learning policies were implemented. In panels **A** and **B**, the daily mean ± 95% CI is shown for different parts of the semester. In panels **C** and **D**, open circles indicate non-class days. In panel **E**, buildings are colour-coded by the daily average of clustering duration. Buildings with missing or incomplete Wi-Fi data are coloured grey.

Next, we evaluated the impact of e-learning on student clustering behaviour, defined as >25 students connected to the same Wi-Fi access point. We surveyed several thousand Wi-Fi access points to determine the number of sites with student clustering and the duration of clustering at each of these sites (**S4 Fig**). There were several hundred Wi-Fi access points where student clustering occurred, with 20% of these sites accounting for about 80% of the total duration of clustering behaviour over the semester (**S5 Fig**). The daily rhythm in number of students on campus drove the pattern of clustering behaviour (**Fig 2A**). In the early part of the semester, student clustering tracked the timing of lectures, whereas this pattern was flattened after e-learning was implemented (**Fig 2B**).

During normal in-class learning, there were about 150 Wi-Fi access points per day where a student cluster was detected (**Fig 2C**), contributing to about 300 hours of clustering behaviour (**Fig 2D**). The transition to e-learning for classes with >50 students was associated with a 70% decrease in the number of sites with a student cluster, as well as the duration of clustering at these sites. These findings differ from the prior academic year, in which student clustering behaviour on school days changed little over the semester (**Fig 2C and 2D**). The transition to e-learning for classes with >25 students effectively eliminated student clustering. These findings were further visualised by plotting the data on a university map to identify hot spots of clustering activity (**Fig 2E**). After e-learning was implemented, there was a marked reduction in student clustering in buildings where students usually converged for classes and social activities.

**Fig 3:**
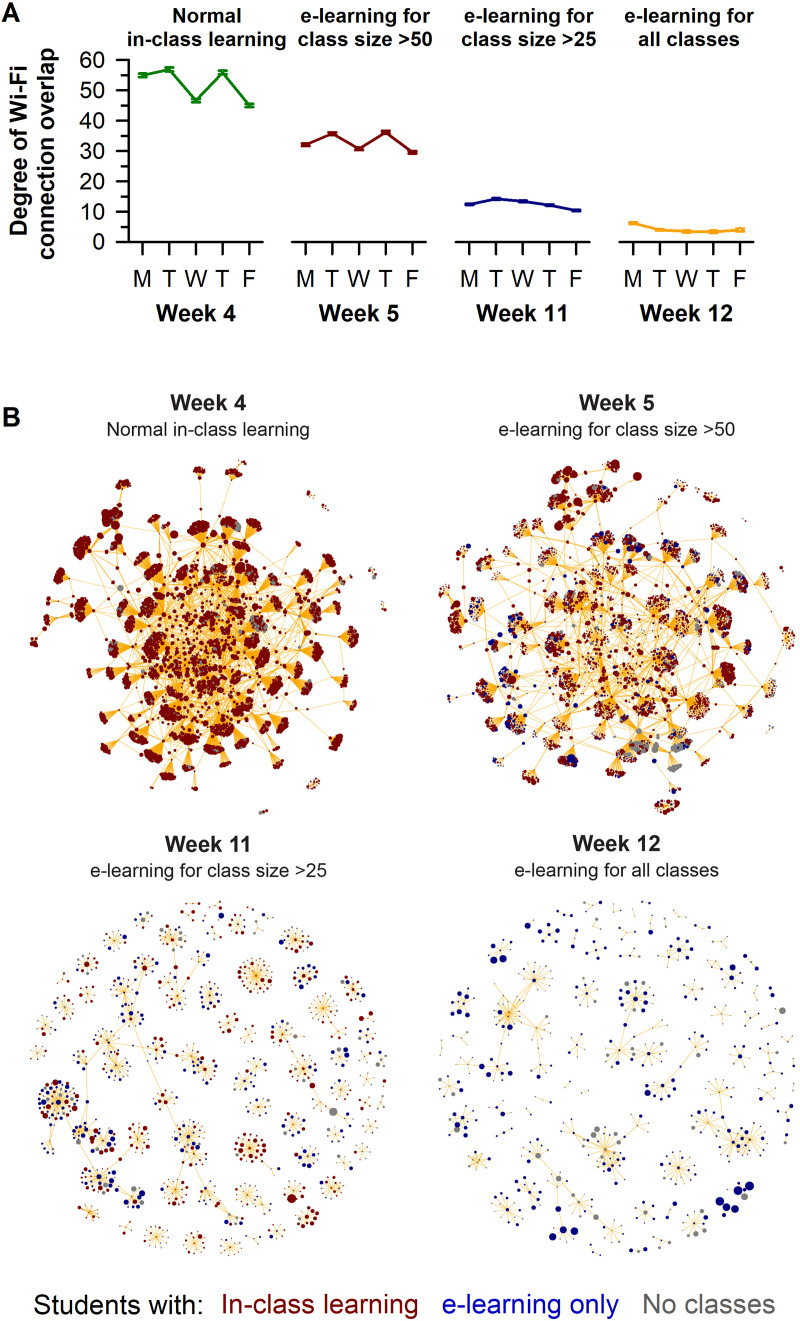
E-learning interventions reduced the number of pairs of students with spatiotemporal overlap during the COVID-19 outbreak. (**A**) Each e-learning intervention was associated with a decrease in the degree of Wi-Fi connection overlap per student. (**B**) Network plots for 100 randomly selected students show that more stringent e-learning policies resulted in a sparser network structure with smaller clusters of students. In panel **A**, the mean ± 95% CI is shown. In panel **B**, the size of each circle relates to the daily duration of time connected to Wi-Fi. The thickness of the orange lines corresponds to the duration of spatiotemporal overlap between pairs of students. Data in the network plots correspond to Mondays for each of the representative weeks with different e-learning policies.

Each e-learning transition was associated with a decrease in the degree of overlap for individual students (i.e., the number of unique pairs formed by a student with others) who connected to the Wi-Fi network (**Fig 3A**). During normal in-class learning, students showed spatiotemporal overlap with about 50 of their peers per day on average. The degree of student overlap dropped by about 30% after the transition to e-learning for classes with >50 students, and by an additional 50% after e-learning was implemented for classes with >25 students. After all classes were delivered by e-learning, students detected on campus overlapped with only about 5 of their peers per day. Weakening of the spatiotemporal student network with each e-learning transition was further visualized by network plots, demonstrating a decrease in both clustering and the degree of Wi-Fi connection overlap between students as more stringent e-learning policies were implemented (**Fig 3B**).

**Fig 4:**
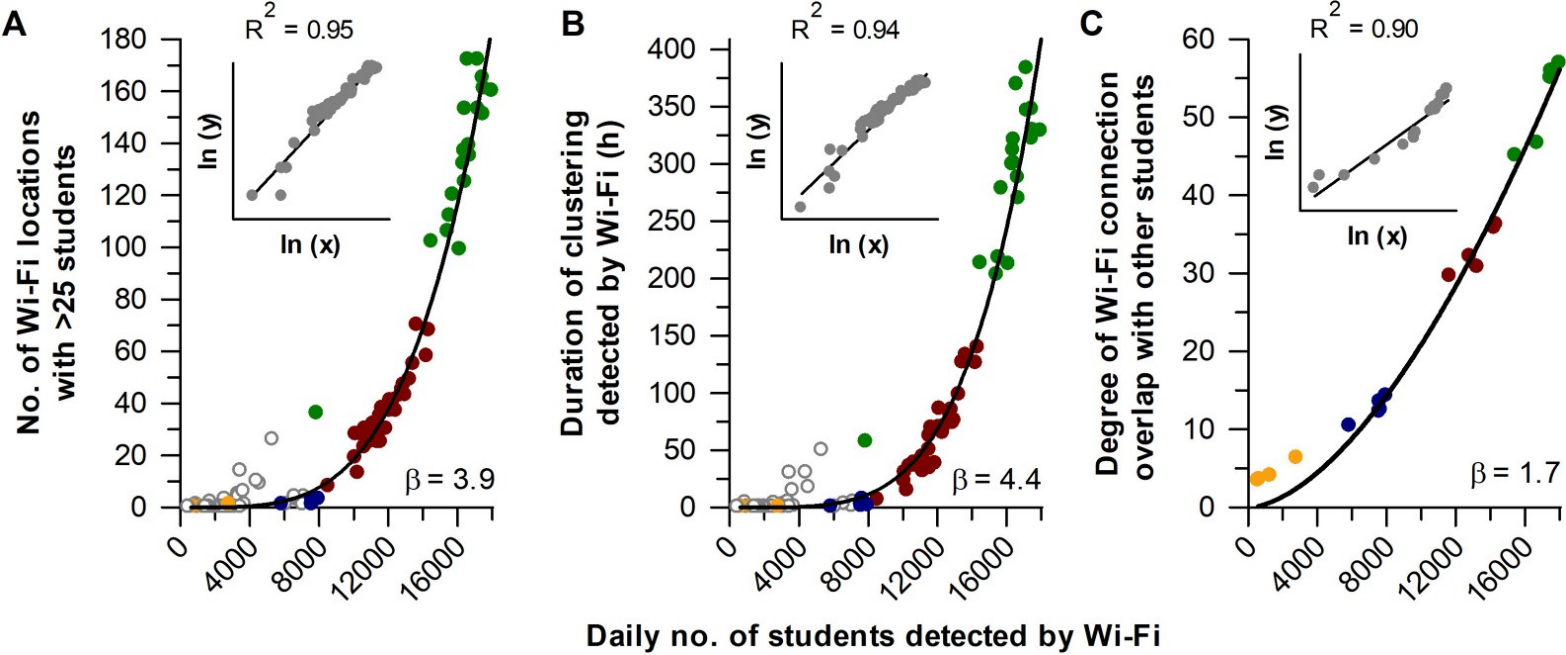
Student clustering showed power law scaling with the number of students on campus. Student mixing showed accelerated growth with daily population size, including (**A**) the number of Wi-Fi locations with a student cluster, (**B**) the duration of student clustering, and (**C**) the degree of spatiotemporal overlap of students with their peers. Data are shown for the second semester of the 2019/20 school year during the COVID-19 outbreak. Each dataset was fitted with a power law function, with β representing the scaling exponent. Insets show results for linear regression after taking the natural logarithm of each variable. Circle colours correspond to different parts of the semester with normal in-class learning (green), e-learning for classes with >50 students (red), e-learning for classes with >25 students (blue), and e-learning for all classes (orange). In panels **A** and **B**, open circles indicate non-class days.

Next, we investigated scaling properties of student mixing patterns with the number of students detected on campus. The number of Wi-Fi access points with student clustering increased with student population size according to a power law distribution (**Fig 4A**). The relationship was super-linear whereby growth in the number of student clusters accelerated with larger numbers of students on campus. Similar results were observed for the daily duration of student clustering (**Fig 4B**). These findings were reproducible using data from the prior academic year, demonstrating that scaling properties of student clustering behaviour with population size were generalisable and not related to the COVID-19 pandemic or implementation of e-learning (**S6 Fig**). Power law scaling of student clustering behaviour was also observed for different types of locations on campus including teaching and non-teaching facilities (**S7 Fig**). Moreover, power law scaling was observed when alternative definitions of cluster size were tested, ranging from >5 to >50 students detected at the same Wi-Fi access point (**S8 Fig**). These analyses showed that larger clusters of students were more sensitive to changes in population size (i.e., the exponent of the power law function was greater), and that e-learning resulted in a marked decrease in the frequency and duration of clustering behaviour for all cluster sizes. In line with these observations, students’ degree of Wi-Fi connection overlap with their peers also exhibited super-linear scaling with daily population size (**Fig 4C**).

## Discussion

Our study is the first to characterise university-wide student mixing patterns during a pandemic. We exploited a natural experiment to test the impact of a phased transition to e-learning on population dynamics at a large university. In doing so, we generated key findings that can be used to make evidence-based decisions on providing educational continuity during a pandemic. Taking into account students’ class schedules and timetables, we show that daily population size can be manipulated in a predictable manner by implementing e-learning for all classes that exceed a given class size. Critically, a small decrease in student population size resulted in a large reduction in student clustering behaviour according to a power law function. This effect was observed by converting a relatively small number of large classes to e-learning, while leaving the vast majority of classes unperturbed. The scaling properties of student mixing patterns suggest that a targeted reduction in student clustering behaviour can be achieved by using e-learning to control the number of students on campus. These findings have important implications for strategies that seek to minimise student-to-student contact during a disease outbreak.

The present study was motivated by the need to provide university decision-makers with feedback on the impact of their e-learning policies on student population dynamics during the pandemic. The analyses were performed and shared with university leaders during the period of time when they were planning how to re-open the university in the following semester (i.e., August, 2020). Taking into consideration student data collected during the pandemic, as well as the improving COVID-19 situation in Singapore, NUS re-opened in the Fall semester with mandatory e-learning for all classes with >50 students. A university-wide contact tracing platform was also implemented based on student/staff connections to the Wi-Fi network. These courses of action confirm that Wi-Fi connection data can be used to track student behaviour and guide a university’s pandemic response. Notably, no known cases of COVID-19 transmission occurred on the NUS campus. While this precluded analyses on the impact of e-learning policies on disease transmission rates, our findings demonstrate that opportunities for student mixing were markedly reduced.

Our study took advantage of the university’s existing Wi-Fi network infrastructure to measure campus-wide spatiotemporal mixing of students. At its core, this method requires counting of students connected to different Wi-Fi access points. Students are only detected if they have a Wi-Fi enabled device that is actively scanning for a Wi-Fi access point. The location where a student is connected also depends on the proximity and range of the nearest Wi-Fi access point. Therefore, it was not possible in our study to estimate the physical distance between students detected at the same Wi-Fi access point. Despite these limitations, prior studies have shown that Wi-Fi connections are as accurate as dedicated physical sensors (e.g., infrared beam-break or thermal sensors) for estimating student occupancy of university rooms and buildings [34, 35]. The daily pattern of student Wi-Fi connections also conforms to expectations for different sites on campus including teaching spaces, libraries, food courts, and residential buildings [34, 36–38], demonstrating that university Wi-Fi networks can be used to monitor students’ use of university resources.

In the present study, Wi-Fi connection data were collected passively without the need for students’ active participation. This enabled us to analyse data across all undergraduate students who connected to the university’s Wi-Fi network (i.e. in 99.5% of students enrolled in classes). There are other approaches for measuring student interactions including Bluetooth proximity detection and direct observation, which can provide more detailed information on student social networks. However, it was not possible to implement those approaches in our study given the pandemic response timeline and practical considerations. Another limitation of our study is that we did not investigate student mixing with university staff or visitors because we only had Wi-Fi connection data for students. In future work, it will be important to evaluate the scaling properties of clustering behaviour while considering all people on campus.

The power law scaling we observed for student mixing behaviour with population size is consistent with prior work demonstrating accelerated growth of human interactions with city population size [21–25]. Epidemiological models indicate that these scaling relationships drive super-linear growth of disease transmission rates as cities get bigger [22, 24, 25]. Like cities, universities are complex systems composed of different infrastructural and social elements whose hierarchical structures give rise to scaling laws [21, 39]. However, we found that the growth rates of student mixing patterns on campus (determined by *β*, the exponent of the power law scaling function) were greater compared with studies on scaling of human interactions with city size. This may be related to differences in student network dynamics and university infrastructural components compared with cities in which they reside. Earlier work found that that *β* ranged from 1.05 to 1.20 for social connectivity patterns derived from mobile phone call records and internet interactions [24, 25]. In those studies, degrees of connectivity were calculated based on direct communication between users. In contrast, students in our study were not necessarily in close proximity when connected to the same Wi-Fi access point. This may have led to an overestimation in the number of student interactions with increasing population size, resulting in a higher scaling exponent for degrees of Wi-Fi connection overlap (*β* = 1.7) compared with previous studies of communication networks. Notably, the scaling exponents for student clustering behaviour were even higher (range, *β* = 1.3 to 5.7), demonstrating a marked acceleration in the number of clusters with increasing population size. Additional studies are needed to understand the factors giving rise to these scaling relationships, and whether they are generalizable to other universities.

During the COVID-19 pandemic, many universities adopted a hybrid learning model in which students took a combination of in-person and online classes [15]. Additional research is needed to determine whether this approach was effective in mitigating the spread of COVID-19. Likewise, the relative impact of school closures on incidence of COVID-19 is uncertain [14]. We found, however, that converting a small number of classes to e-learning sharply reduced student mixing patterns across the entire university campus. This would be expected to decrease the potential for disease transmission. Network-based simulation models show that infectious diseases spread from person to person within a community through the social contact network [40]. These networks can be controlled by targeted physical distancing strategies. The present study identifies e-learning as a potential strategy for controlling student contacts within the campus community and reducing disease transmission.

## Conclusions

In conclusion, a targeted decrease in student mixing behaviour during a disease outbreak can be achieved by implementing a partial transition to e-learning. Based on the success of this approach at NUS in providing educational continuity during the pandemic, we recommend that e-learning and monitoring of students’ mixing patterns (e.g., using Wi-Fi connection data) be incorporated into each university’s pandemic preparedness plan. First, universities should evaluate their daily class size distribution to determine the impact of a given e-learning policy on the number of students with in-class learning. This information makes it possible to achieve a targeted reduction in student population size because the number of students on campus is dependent on the proportion of students with in-class learning. Second, universities should develop the capability to count the number of students on campus because population size is a main driver of student clustering behaviour and mixing patterns. We showed that this can be achieved using existing Wi-Fi network infrastructure, and the data can be used to derive scaling properties of student mixing with population size. Third, universities should consider how to implement e-learning in view of the local and nationwide health response to a disease outbreak. In the present study, a partial transition to e-learning took place near the start of the pandemic when community spread was low. This approach was implemented a second time during recovery to normal school operations. A full transition to e-learning may be necessary during periods of high community disease transmission, especially if government mandated stay-at-home orders are in place. Taken together, our study establishes a roadmap that universities can follow for making evidence-based decisions on students’ learning and safety during the COVID-19 pandemic and future disease outbreaks.

## Supporting information

S1 Fig Students detected on campus during normal university operations. Data are shown for the second semester of the 2018/19 school year at the National University of Singapore (NUS), assessed one year before the COVID-19 outbreak. The number of students per day who connected to the NUS Wi-Fi network is shown for (**A**) the entire campus and (**B**) different types of locations on campus. (**C**) The daily percentage of students detected by Wi-Fi was about two-fold greater in students with in-class learning versus no scheduled class. In panels **B** and **C**, open circles indicate non-class days.
(TIF)

S2 Fig Class size characteristics at the National University of Singapore (NUS). (**A**) The distribution of class sizes is shown for the second semester of the 2019/20 school year in which the COVID-19 outbreak occurred. Class sizes were categorised as small (green; ≤25 students), medium (blue; >25 to ≤50 students), or large (red; >50 students). (**B**) The combined student enrolment in medium and large classes was greater than enrolment in small classes. (**C**) The cumulative distribution plot shows the number of students whose smallest class of the day exceeded a given class size threshold. The black trace with shaded grey lines shows the daily mean and range. The red dropline shows that the transition to e-learning for classes with >50 students resulted in about 5,000 students per day who had classes delivered only by e-learning. The blue dropline shows that the transition to e-learning for classes with >25 students resulted in about 9,000 students per day who had classes delivered only by e-learning. When all classes were shifted to e-learning there were about 18,000 students per day taking their classes online.
(TIF)

S3 Fig The daily number of students detected on campus was predicted by the number of students with in-class learning. Data are shown for the second semester of the 2019/20 school year at the National University of Singapore (NUS) during the COVID-19 outbreak. The number of students per day who connected to the NUS Wi-Fi network is plotted against the daily number of students who had at least one class session that took place on campus. Circle colours correspond to different parts of the semester with normal in-class learning (green), e-learning for classes with >50 students (red), e-learning for classes with >25 students (blue), and e-learning for all classes (orange). The solid black trace shows the best-fit linear regression model, and the dashed black trace is the unity line.
(TIF)

S4 Fig Campus-wide detection of students at different Wi-Fi access points at the National University of Singapore (NUS). The daily peak in the number of students who connected to each Wi-Fi access point is shown for (**A**) the second semester of the 2018/19 school year, and (**B**) the second semester of the 2019/20 school year in which the COVID-19 outbreak occurred. Each peak value corresponds to largest number of students per day detected at a given Wi-Fi access point over a 15-min period. Each row in the heat map represents a different Wi-Fi access point with green and magenta colours indicating the number of students who were detected.
(TIF)

S5 Fig Distribution of student clustering across Wi-Fi access points at the National University of Singapore (NUS). The cumulative duration of student clustering (>25 students connected to the same Wi-Fi access point) is shown for (**A**) the second semester of the 2018/19 school year, and (**B**) the second semester of the 2019/20 school year in which the COVID-19 outbreak occurred. Data are plotted for Wi-Fi access points with at least one student cluster detected during the semester (785 out of 6,573 locations in 2018/19; 564 out of 6,313 locations in 2019/20). Wi-Fi access points in each plot are ordered from left to right by the cumulative duration of student clustering over the entire semester.
(TIF)

S6 Fig Student clustering showed power law scaling with the number of students on campus. Students’ Wi-Fi connection data were analysed for the second semester of the 2018/19 school year and compared with the second semester of the 2019/20 school year in which the COVID-19 outbreak occurred. In both semesters, student clustering behaviour showed accelerated growth with increasing number of students detected on campus, including (**A**) the number of Wi-Fi locations with a student cluster (>25 students connected to the same Wi-Fi access point), and (**B**) the duration of student clustering at these locations. Each dataset was fitted with a power law function, with β representing the scaling exponent. Insets show results for linear regression after taking the natural logarithm of each variable for the 2018/19 school year. Filled circles show school days and open circles indicate non-class days.
(TIF)

S7 Fig Student clustering at different campus locations showed power law scaling with the number of students detected on campus. Data are shown for the second semester of the 2019/20 school year at the National University of Singapore (NUS) during the COVID-19 outbreak. In both (**A**) teaching facilities and (**B**) non-teaching facilities, the number of Wi-Fi locations with >25 students (left panels) and the duration of clustering behaviour (right panels) showed accelerated growth with increasing number of students detected on campus. Each dataset was fitted with a power law function, with β representing the scaling exponent. Insets show results for linear regression after taking the natural logarithm of each variable. Circle colours correspond to different parts of the semester with normal in-class learning (green), e-learning for classes with >50 students (red), e-learning for classes with >25 students (blue), and e-learning for all classes (orange). Open circles indicate non-class days.
(TIF)

S8 Fig Power law scaling of different student cluster sizes with number of students detected on campus. Data are shown for the second semester of the 2019/20 school year at the National University of Singapore (NUS) during the COVID-19 outbreak. Different definitions of a student cluster were tested ranging from >5 to >50 students detected at the same Wi-Fi access point. For all cluster sizes, student clustering behaviour showed accelerated growth with increasing number of students detected on campus, including (**A**) the number of Wi-Fi locations with a student cluster, and (**B**) the duration of student clustering at these locations. Each dataset was fitted with a power law function, with β representing the scaling exponent. Insets show results for linear regression after taking the natural logarithm of each variable. Circle colours correspond to different parts of the semester with normal in-class learning (green), e-learning for classes with >50 students (red), e-learning for classes with >25 students (blue), and e-learning for all classes (orange). Open circles indicate non-class days.
(TIF)

S1 Table University policies and advisories during the COVID-19 outbreak. (DOCX)

S2 Table Student characteristics and general information. (DOCX)
