# Supplementary figures and images for: A targeted e-learning approach for keeping universities open during the COVID-19 pandemic while reducing student physical interactions
Source: PLoS One. 2021 Apr 8;16(4):e0249839. doi: 10.1371/journal.pone.0249839 (PMC8031760; doi:10.1371/journal.pone.0249839)

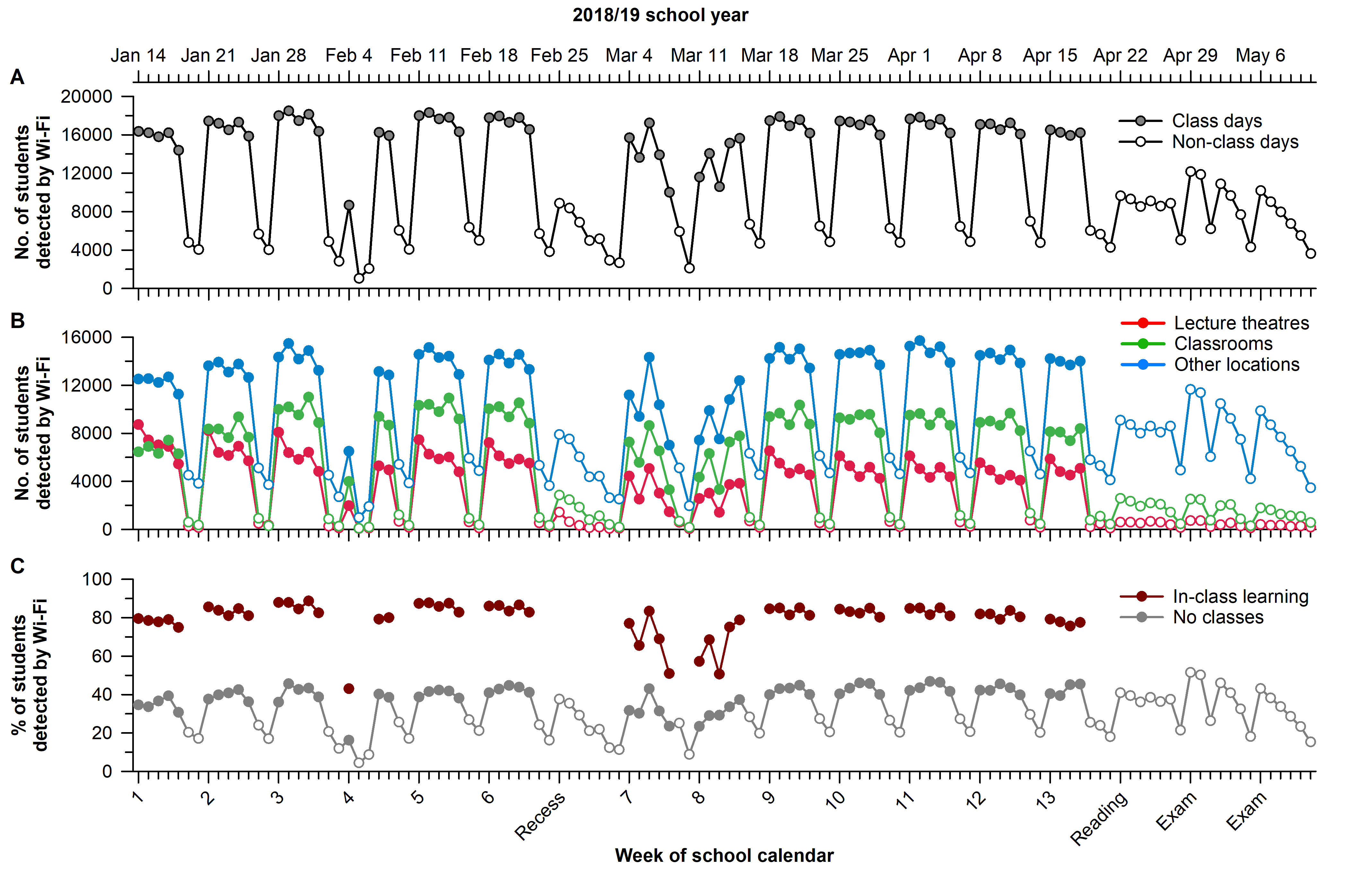

Supplement: S1 Fig — Data are shown for the second semester of the 2018/19 school year at the National University of Singapore (NUS), assessed one year before the COVID-19 outbreak. The number of students per day who connected to the NUS Wi-Fi network is shown for (A) the entire campus and (B) different types of locations on campus. (C) The daily percentage of students detected by Wi-Fi was about two-fold greater in students with in-class learning versus no scheduled class. In panels B and C, open circles indicate non-class days. (TIF) [file pone.0249839.s001.tif]

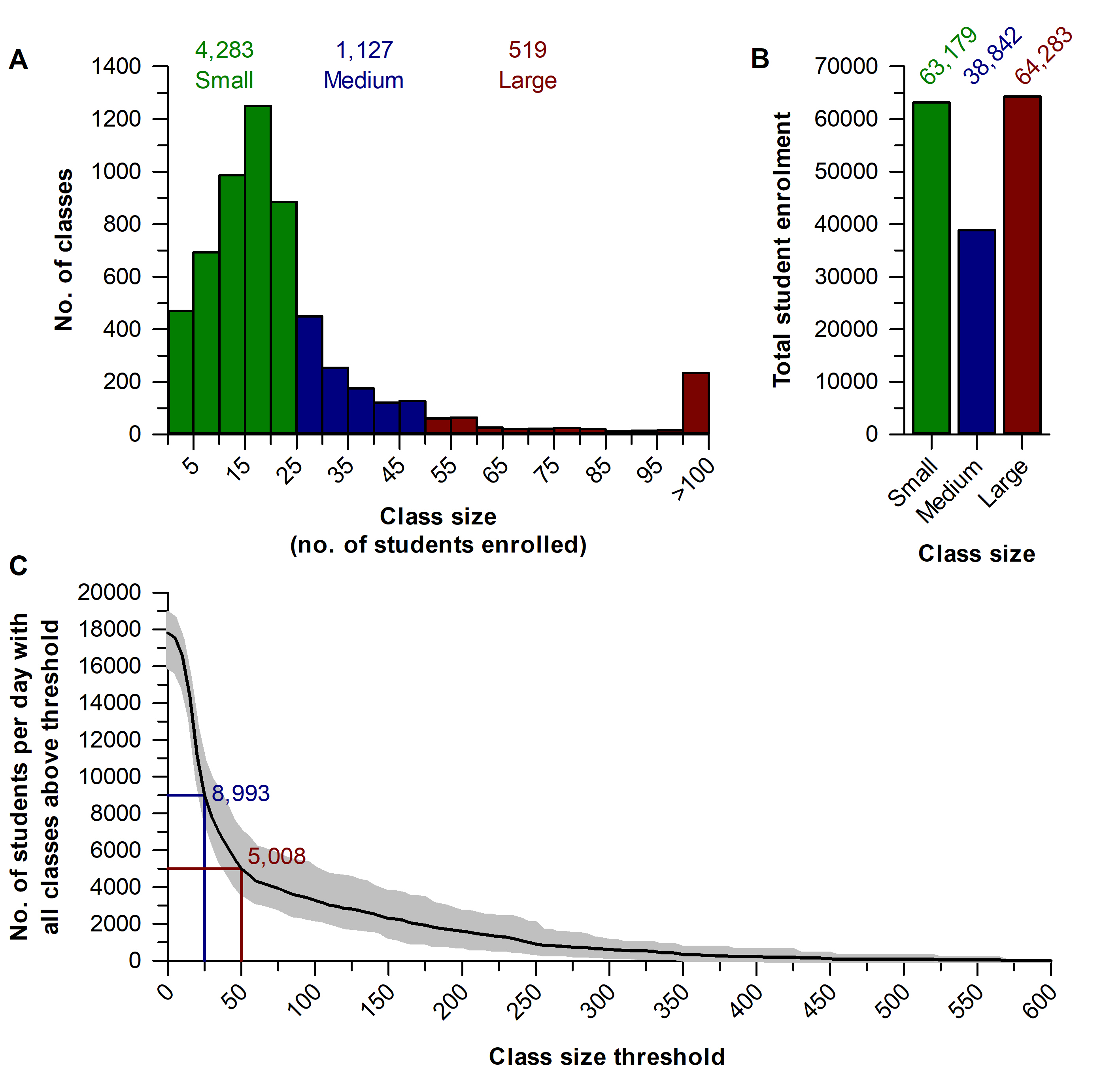

Supplement: S2 Fig — (A) The distribution of class sizes is shown for the second semester of the 2019/20 school year in which the COVID-19 outbreak occurred. Class sizes were categorised as small (green; ≤25 students), medium (blue; >25 to ≤50 students), or large (red; >50 students). (B) The combined student enrolment in medium and large classes was greater than enrolment in small classes. (C) The cumulative distribution plot shows the number of students whose smallest class of the day exceeded a given class size threshold. The black trace with shaded grey lines shows the daily mean and range. The red dropline shows that the transition to e-learning for classes with >50 students resulted in about 5,000 students per day who had classes delivered only by e-learning. The blue dropline shows that the transition to e-learning for classes with >25 students resulted in about 9,000 students per day who had classes delivered only by e-learning. When all classes were shifted to e-learning there were about 18,000 students per day taking their classes online. (TIF) [file pone.0249839.s002.tif]

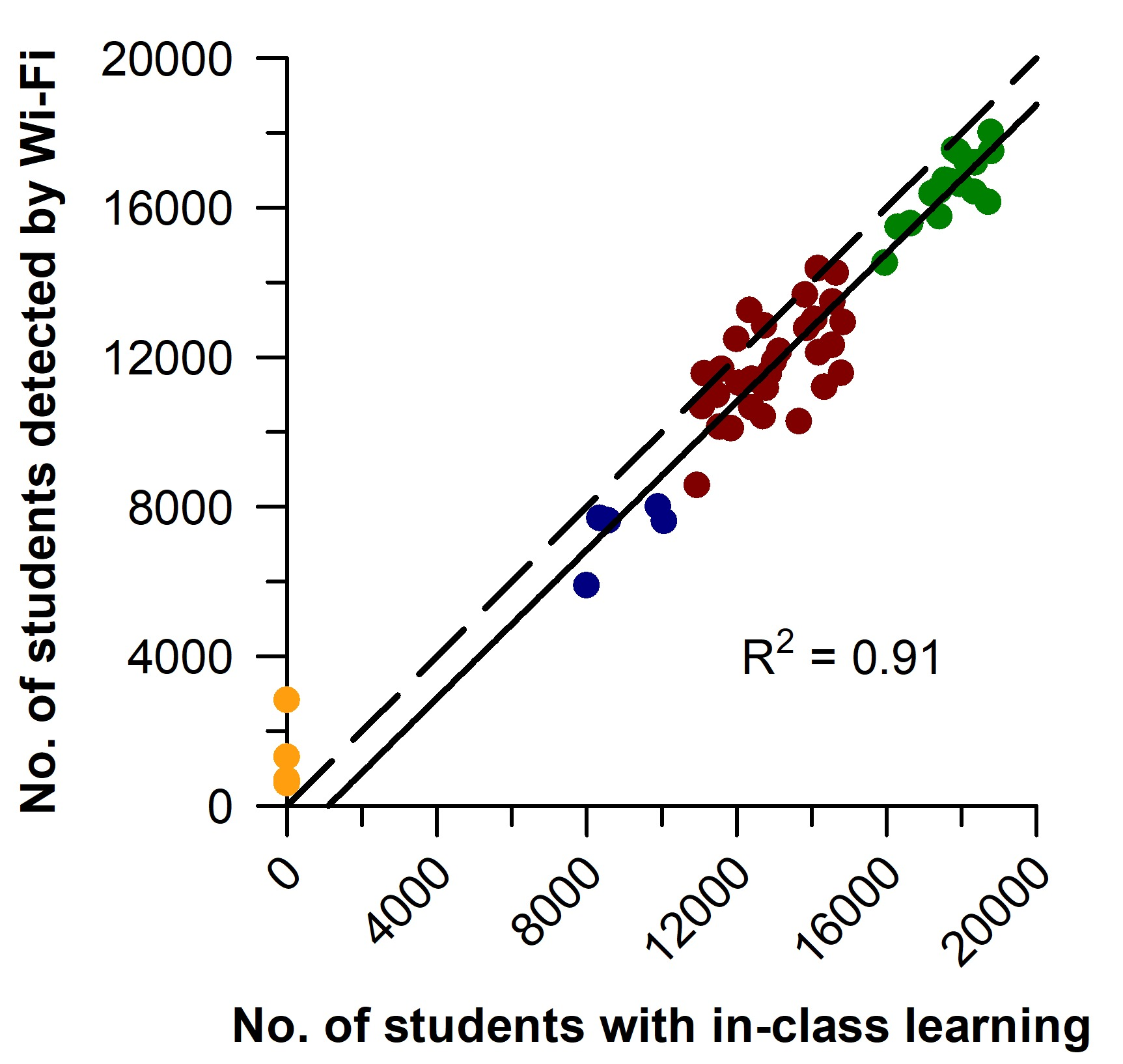

Supplement: S3 Fig — Data are shown for the second semester of the 2019/20 school year at the National University of Singapore (NUS) during the COVID-19 outbreak. The number of students per day who connected to the NUS Wi-Fi network is plotted against the daily number of students who had at least one class session that took place on campus. Circle colours correspond to different parts of the semester with normal in-class learning (green), e-learning for classes with >50 students (red), e-learning for classes with >25 students (blue), and e-learning for all classes (orange). The solid black trace shows the best-fit linear regression model, and the dashed black trace is the unity line. (TIF) [file pone.0249839.s003.tif]

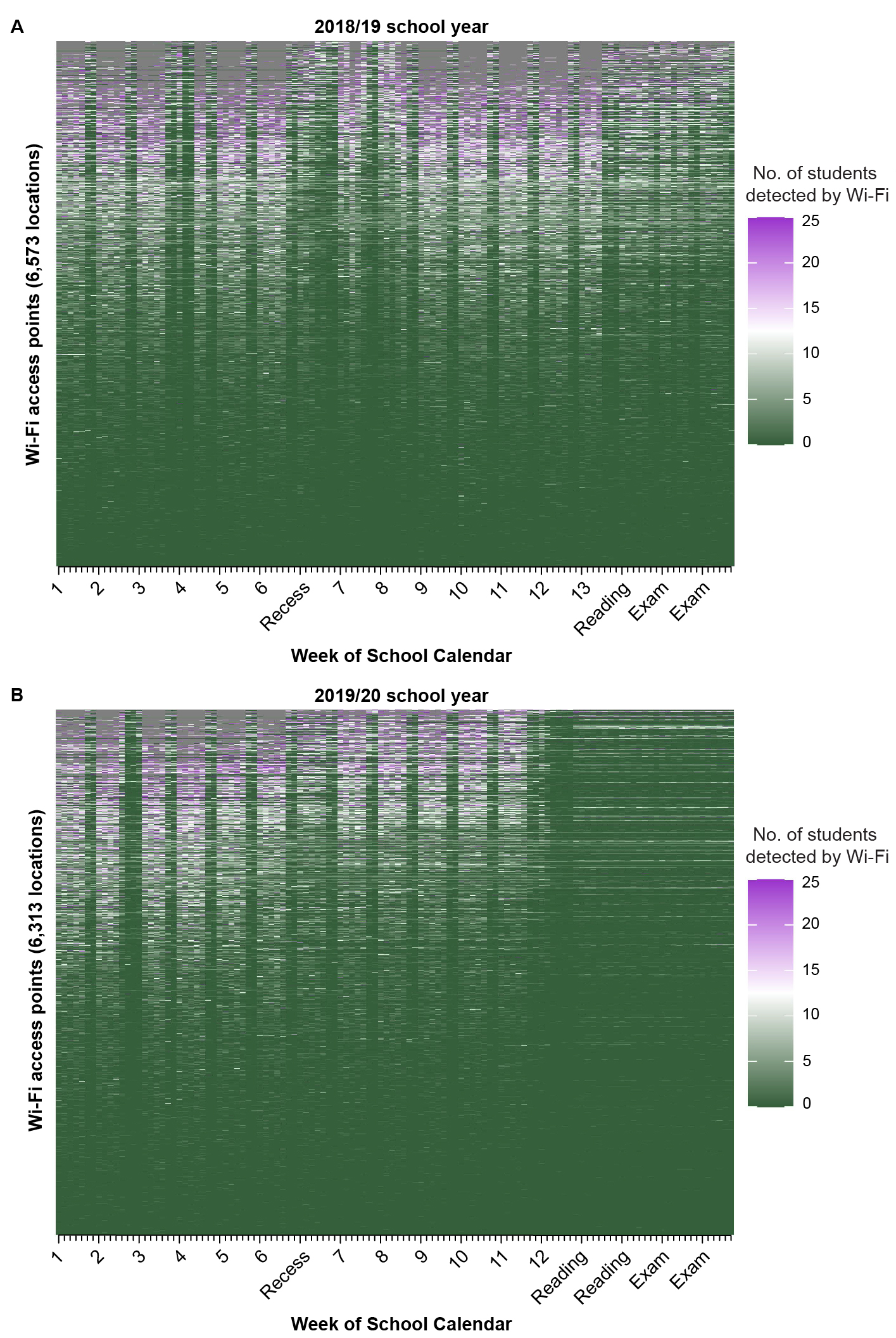

Supplement: S4 Fig — The daily peak in the number of students who connected to each Wi-Fi access point is shown for (A) the second semester of the 2018/19 school year, and (B) the second semester of the 2019/20 school year in which the COVID-19 outbreak occurred. Each peak value corresponds to largest number of students per day detected at a given Wi-Fi access point over a 15-min period. Each row in the heat map represents a different Wi-Fi access point with green and magenta colours indicating the number of students who were detected. (TIF) [file pone.0249839.s004.tif]

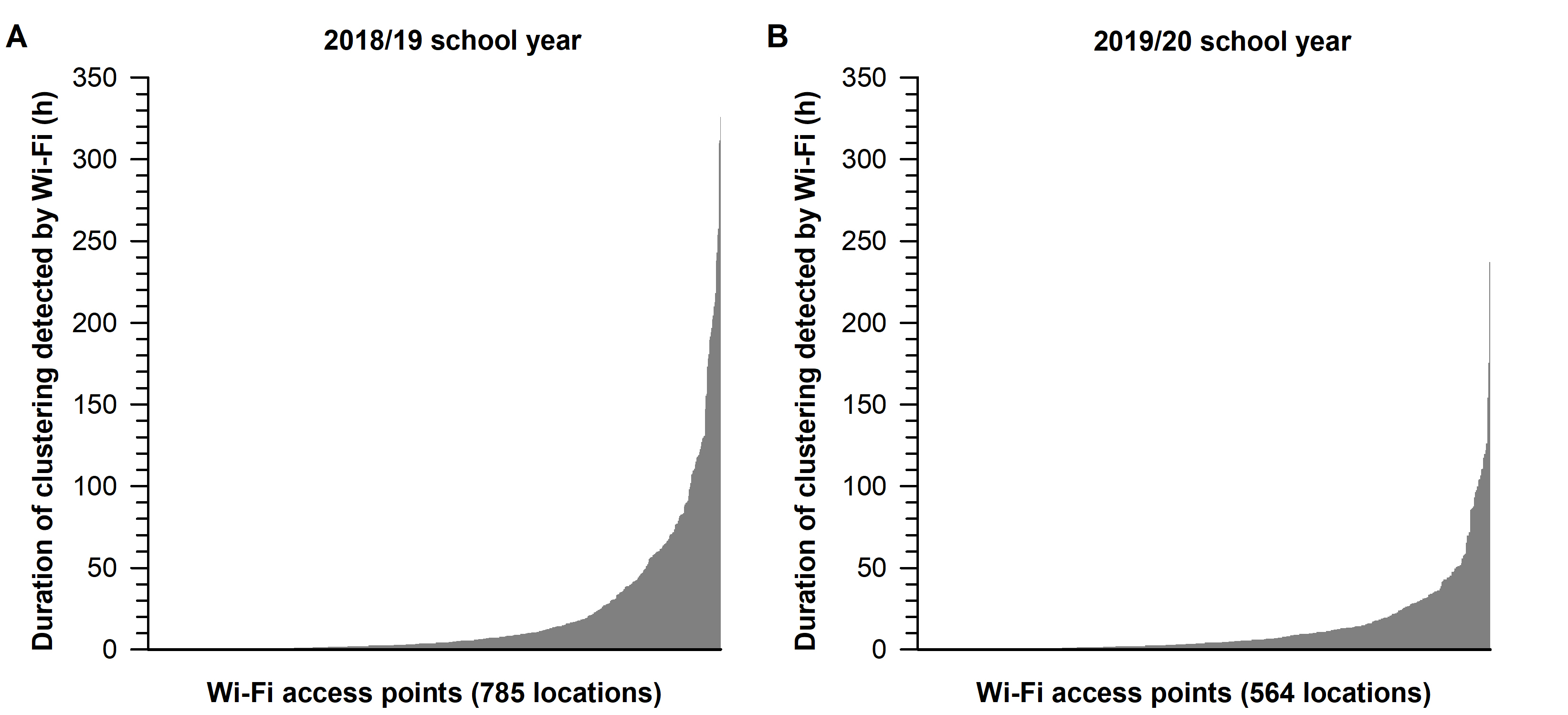

Supplement: S5 Fig — The cumulative duration of student clustering (>25 students connected to the same Wi-Fi access point) is shown for (A) the second semester of the 2018/19 school year, and (B) the second semester of the 2019/20 school year in which the COVID-19 outbreak occurred. Data are plotted for Wi-Fi access points with at least one student cluster detected during the semester (785 out of 6,573 locations in 2018/19; 564 out of 6,313 locations in 2019/20). Wi-Fi access points in each plot are ordered from left to right by the cumulative duration of student clustering over the entire semester. (TIF) [file pone.0249839.s005.tif]

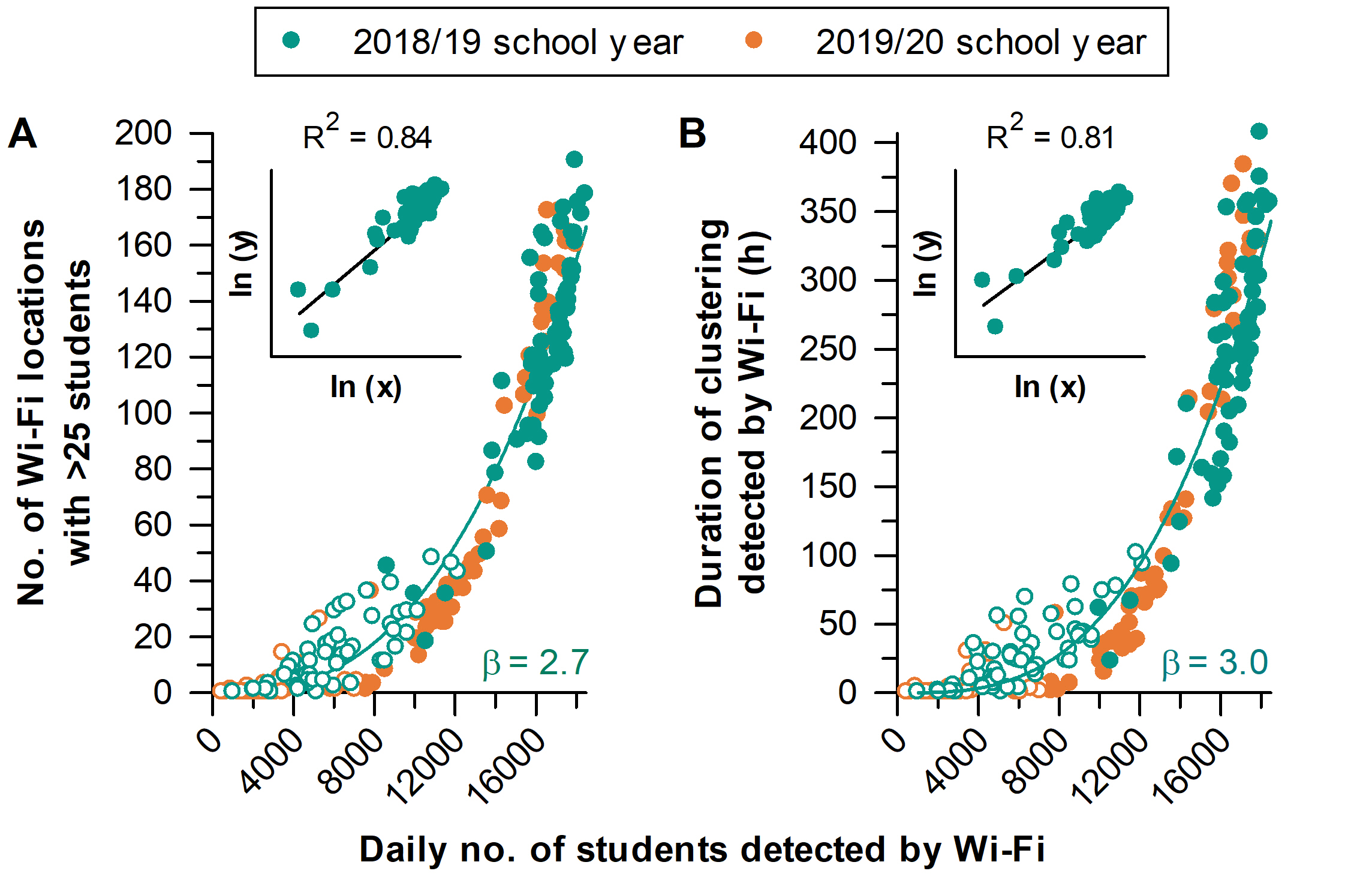

Supplement: S6 Fig — Students’ Wi-Fi connection data were analysed for the second semester of the 2018/19 school year and compared with the second semester of the 2019/20 school year in which the COVID-19 outbreak occurred. In both semesters, student clustering behaviour showed accelerated growth with increasing number of students detected on campus, including (A) the number of Wi-Fi locations with a student cluster (>25 students connected to the same Wi-Fi access point), and (B) the duration of student clustering at these locations. Each dataset was fitted with a power law function, with β representing the scaling exponent. Insets show results for linear regression after taking the natural logarithm of each variable for the 2018/19 school year. Filled circles show school days and open circles indicate non-class days. (TIF) [file pone.0249839.s006.tif]

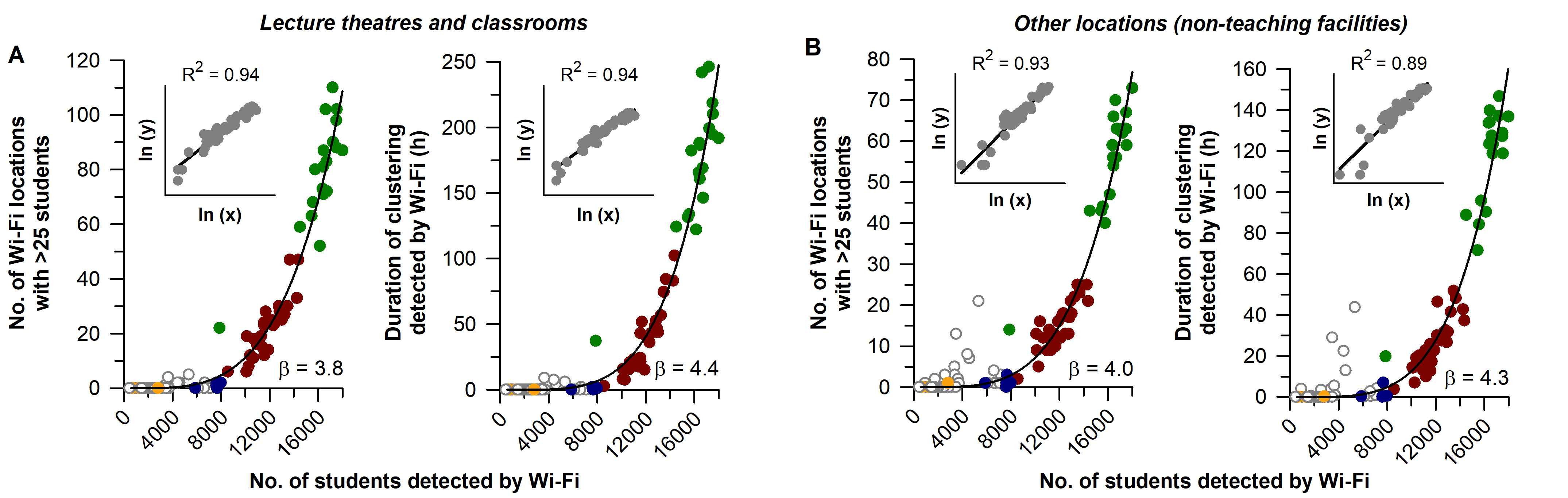

Supplement: S7 Fig — Data are shown for the second semester of the 2019/20 school year at the National University of Singapore (NUS) during the COVID-19 outbreak. In both (A) teaching facilities and (B) non-teaching facilities, the number of Wi-Fi locations with >25 students (left panels) and the duration of clustering behaviour (right panels) showed accelerated growth with increasing number of students detected on campus. Each dataset was fitted with a power law function, with β representing the scaling exponent. Insets show results for linear regression after taking the natural logarithm of each variable. Circle colours correspond to different parts of the semester with normal in-class learning (green), e-learning for classes with >50 students (red), e-learning for classes with >25 students (blue), and e-learning for all classes (orange). Open circles indicate non-class days. (TIF) [file pone.0249839.s007.tif]

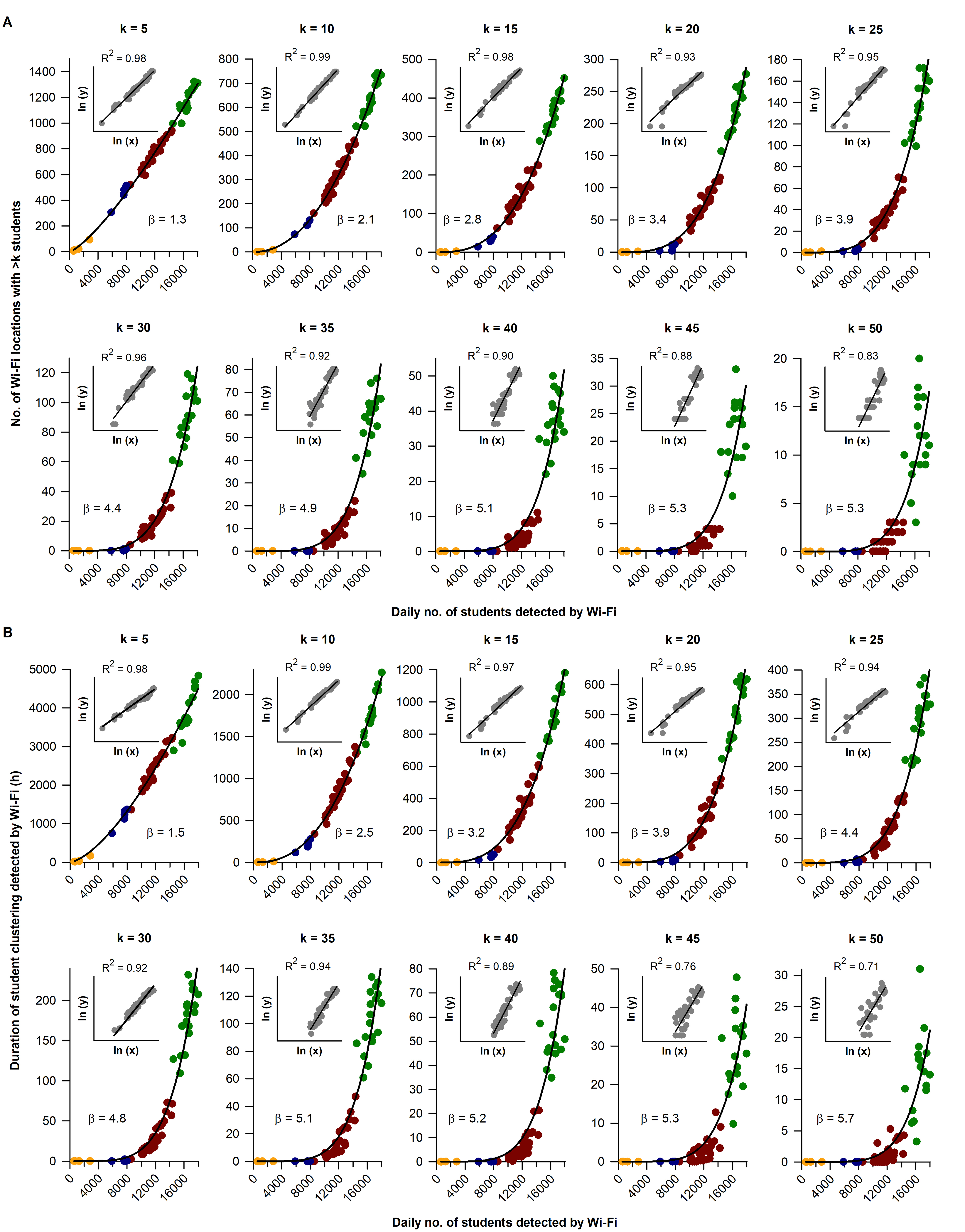

Supplement: S8 Fig — Data are shown for the second semester of the 2019/20 school year at the National University of Singapore (NUS) during the COVID-19 outbreak. Different definitions of a student cluster were tested ranging from >5 to >50 students detected at the same Wi-Fi access point. For all cluster sizes, student clustering behaviour showed accelerated growth with increasing number of students detected on campus, including (A) the number of Wi-Fi locations with a student cluster, and (B) the duration of student clustering at these locations. Each dataset was fitted with a power law function, with β representing the scaling exponent. Insets show results for linear regression after taking the natural logarithm of each variable. Circle colours correspond to different parts of the semester with normal in-class learning (green), e-learning for classes with >50 students (red), e-learning for classes with >25 students (blue), and e-learning for all classes (orange). Open circles indicate non-class days. (TIF) [file pone.0249839.s008.tif]
