# Supplementary material for: A targeted e-learning approach for keeping universities open during the COVID-19 pandemic while reducing student physical interactions
Source: PLoS One. 2021 Apr 8;16(4):e0249839. doi: 10.1371/journal.pone.0249839 (PMC8031760; doi:10.1371/journal.pone.0249839)
Supplement: S1 Table — (DOCX) [file pone.0249839.s009.docx]

**S1 Table.** **University policies and advisories during the COVID-19 outbreak**

| **Date** | **National University of Singapore (NUS) policies and advisories during the COVID-19 outbreak** |
| --- | --- |
| 23-Jan-2020 | Advisory to defer travel plans to China with immediate effect |
| 27-Jan-2020 | Leave of absence for 14 days for students/staff returning to Singapore from China; Daily online reporting of body temperature for students/staff staying in NUS hostels |
| 29-Jan-2020 | Mandatory online reporting of planned overseas travel; Quarantine orders for students/staff with recent travel to Hubei province, China; NUS staff/student identification card required to access NUS units |
| 31-Jan-2020 | Summary of disciplinary actions for breaching leave of absence measures; Visitor registration required to enter NUS units to facilitate contact tracing |
| 2-Feb-2020 | Updates on students who have been served quarantine orders and leave of absence |
| 8-Feb-2020 | E-learning for classes with >50 students; Events and activities with >50 students cancelled or postponed; Daily online reporting of body temperature for all students/staff; Body temperature screening at all NUS buildings; All policies take effect from 10-Feb-2020 |
| 9-Feb-2020 | Updates and clarifications on daily body temperature reporting and temperature screening on NUS campus |
| 10-Feb-2020 | Meal services provided only to patrons with a NUS identification card or visitors who have completed body temperature screening |
| 20-Feb-2020 | Suspension of co-curricular activities and related events involving close contact |
| 21-Feb-2020 | Advisory on national stay-home notice for persons with recent travel to China; Mandatory online updating of overseas travel and plans |
| 27-Feb-2020 | Advisory on precautionary measures for students/staff returning to Singapore from Korea, Italy, and Iran; E-learning and physical distancing for 7 days for students returning from these countries |
| 4-Mar-2020 | Advisory on national stay-home notice for persons with recent travel to Korea, Italy, and Iran; Updated precautionary measures for staff/students returning from these countries |
| 9-Mar-2020 | Mandatory online updating of overseas travel and plans; Students who do not comply will be unable to access the NUS learning management system |
| 15-Mar-2020 | Suspension of all overseas placements of students/staff; Advisory to defer all official and non-essential travel; Updated precautionary measures for students/staff returning from overseas |
| 18-Mar-2020 | Extended suspension of co-curricular activities and related events involving close contact; Updated precautionary measures for students/staff returning from overseas; E-learning for 2 weeks for students affected by the Malaysia Movement Control Order |
| 19-Mar-2020 | Update on national advisory to defer overseas travel; Updated precautionary measures for returning travellers; E-learning for 2 weeks for all students returning from overseas |
| 25-Mar-2020 | E-learning for classes with >25 students; Events and activities with >25 students cancelled or postponed; All policies take effect from 30-Mar-2020. |
| 27-Mar-2020 | Restricted access to NUS sports facilities, gyms, and swimming pools |
| 28-Mar-2020 | Revision to the school calendar; Final week of scheduled classes to be replaced with an additional reading week |
| 30-Mar-2020 | Advisory on enhanced physical distancing measures; No more than 25 persons in any NUS venue and at least 1 meter between individuals is required; Updates on safe distancing measures during tests and examinations |
| 31-Mar-2020 | Closure of all sports facilities, gyms, and swimming pools |
| 2-Apr-2020 | Update on business continuity plans for NUS units |
| 4-Apr-2020 | E-learning for all classes; Online assessments for tests and examinations; Students encouraged to move home if they have a residence in Singapore; Students remaining in hostels must stay until end of term to comply with nationwide ‘circuit breaker’ measures; Closure of libraries and dining halls; No social activities permitted on campus; All policies take effect from 7-Apr-2020 |
| 5-Apr-2020 | Students can apply for special permission to study on campus in selected venues if unable to perform e-learning at home |
| 9-Apr-2020 | Advisory on strict adherence to safe distancing measures |
| 15-Apr-2020 | Mandatory wearing of a mask when leaving a person’s place of residence; Only students given prior approval are allowed on campus; Disciplinary action for non-compliance with university and national safe distancing measures |
