# Supplementary material for: A targeted e-learning approach for keeping universities open during the COVID-19 pandemic while reducing student physical interactions
Source: PLoS One. 2021 Apr 8;16(4):e0249839. doi: 10.1371/journal.pone.0249839 (PMC8031760; doi:10.1371/journal.pone.0249839)
Supplement: S2 Table — (DOCX) [file pone.0249839.s010.docx]

**S2 Table.** **Student characteristics and general information**

|  | 2018/19 school year  (*n* = 23,668) | 2019/20 school year  (*n* = 23,993) |
| --- | --- | --- |
| Demographic information |  |  |
| Age in years (mean ± SD) | 21.7 ± 2.1 | 21.6 ± 1.9 |
| Sex, *n* (%) |  |  |
| Female | 12,179 (51.5%) | 12,377 (51.6%) |
| Male | 11,489 (48.5%) | 11,616 (48.4%) |
| Ethnicity, *n* (%) |  |  |
| Chinese | 20,391 (86.2%) | 20,432 (85.2%) |
| Indian | 1,177 (5.0%) | 1,349 (5.6%) |
| Malay | 737 (3.1%) | 778 (3.2%) |
| Others | 1,363 (5.8%) | 1,434 (6.0%) |
| Citizenship, *n* (%) |  |  |
| Singaporean / Permanent Resident | 21,396 (90.4%) | 21,645 (90.2%) |
| Others | 2,272 (9.6%) | 2,348 (9.8%) |
| Class year, *n* (%) |  |  |
| 1 | 7,359 (31.1%) | 7,457 (31.1%) |
| 2 | 6,165 (26.0%) | 6,897 (28.7%) |
| 3 | 4,593 (19.4%) | 4,561 (19.0%) |
| 4 | 5,160 (21.8%) | 4,812 (20.1%) |
| >4 | 391 (1.7%) | 266 (1.1%) |
| Class information |  |  |
| No. of course modules offered | 1,816 | 1,734 |
| No. of class/tutorial groups | 6,104 | 5,929 |
| Wi-Fi connection information |  |  |
| Students who used Wi-Fi, *n* (%) | 23,586 (99.7%) | 23,881 (99.5%) |
| No. of Wi-Fi access points connected | 6,573 | 6,313 |
| No. of Wi-Fi connections | 19,282,203 | 4,871,285 |
